# Magnon Confinement in an All-on-Chip YIG Cavity Resonator Using Hybrid YIG/Py Magnon Barriers

**DOI:** 10.1021/acs.nanolett.3c02388

**Published:** 2023-10-11

**Authors:** Obed Alves Santos, Bart J. van Wees

**Affiliations:** † Physics of Nanodevices Zernike Institute for Advanced Materials, University of Groningen, Nijenborgh 4 AG, Groningen 9747, The Netherlands; ‡ Cavendish Laboratory, University of Cambridge, Cambridge CB3 0HE, United Kingdom

**Keywords:** Magnonics, Spin pumping, Spin waves, YIG resonators, On-chip cavity magnonics

## Abstract

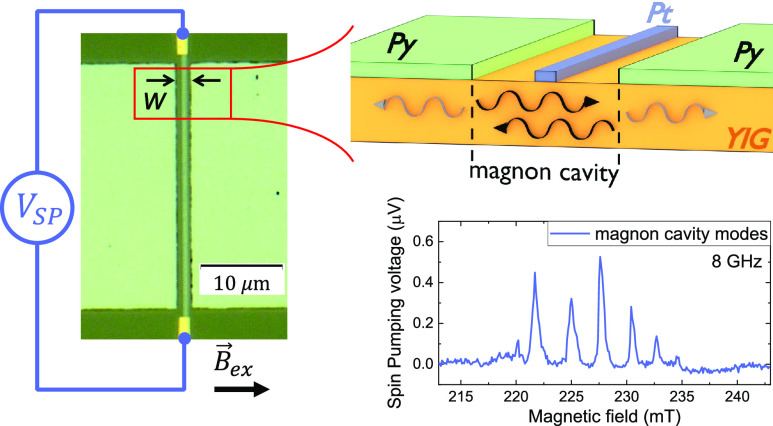

Confining magnons in cavities can introduce new functionalities to magnonic devices, enabling future magnonic structures to emulate the established photonic and electronic components. As a proof-of-concept, we report magnon confinement in a lithographically defined all-on-chip YIG cavity created between two YIG/Permalloy bilayers. We take advantage of the modified magnetic properties of the covered/uncovered YIG film to define on-chip distinct regions with boundaries capable of confining magnons. We confirm this by measuring multiple spin-pumping voltage peaks in a 400 nm wide platinum strip placed along the center of the cavity. These peaks coincide with multiple spin-wave resonance modes calculated for a YIG slab with the corresponding geometry. The fabrication of micrometer-sized YIG cavities following this technique represents a new approach to control coherent magnons, while the spin-pumping voltage in a nanometer-sized Pt strip demonstrates to be a noninvasive local detector of the magnon resonance intensity.

Magnonics aims to transmit, store, and process information in micro- and nanoscale by means of magnons, the quanta of spin waves.^1,2^ Typical studies in this field use the ferrimagnet Yttrium Iron Garnet (Y_3_Fe_5_O_12_, YIG) as a key material, due to its desirable properties, such as very low magnetic losses,^2^ large applicability in mainstream electronics,^3^ long magnon spin relaxation time,^4^ and high magnon spin conductivity.^5,6^ The magnetic proprieties of YIG can be modified by the presence of strong exchange/dipolar coupling caused by the deposition of a ferromagnetic layer onto the YIG film.^7,8^ These hybrid magnonic systems, such as YIG/Py,^8−13^ YIG/CoFeB,^14,15^ and YIG/Co,^16,17^ draw attention from fundamental and applied physics toward the control of coherent magnon excitations for information processing.^18^ Alternatively, systems in which the magnetic material strongly interacts with electrodynamic cavities also provide a good platform for next-generation quantum information technologies, using the dual magnon-photon nature to enable new quantum functionalities.^19,20^ The field called cavity magnonics, among many applications, can offer good compatibility with CMOS, room temperature operation, and GHz-to-THz spin-wave transducers.^21−24^

The possibility of confining magnons in cavities may enable future magnonic devices that emulate established electronic and photonic components, such as magnonic quantum point contacts, magnonic crystals, magnonic quantum bits, magnonic frequency combs, among others.^25−33^ Building upon similar ideas, recent theoretical results suggest all-on-chip structures to produce magnonic cavities by magneto-dipole interaction with a chiral magnonic element,^34^ using an array of antiferromagnets on a ferromagnet,^35^ or by proximity with superconductors.^36^

One approach to confining magnons involves the fabrication of rectangular or circular nano- and microstructures using YIG.^37−40^ Many of these structures are made by etching the YIG film or sputtering YIG from a target on defined microstructures, adding an extra step in the fabrication process and limiting the options of available structures. Recently, Qin et al., demonstrated the (partial) confinement of magnons in a region of the YIG film covered by a ferromagnetic metal.^29^ This confinement arises from the difference in magnetic properties between the covered and uncovered regions of the YIG film, resulting in magnon reflections at the boundaries. Such phenomena enabled the fabrication of an on-chip nanoscale magnonic Fabry–Pérot cavity,^28,29^ observed by the transmission of magnons through the YIG film.^30^

In this paper, we complement these studies by achieving an order of magnitude higher magnon confinement in a YIG film region that is *not covered* by the ferromagnetic metal, preserving the optimal magnetic properties of YIG within the cavity. We report, as a proof-of-concept, the fabrication of an all-on-chip micrometer-sized YIG cavity, by partially covering 100 nm thick LPE-grown YIG film with two square-shaped 30 nm thick permalloy (Py) layers. The exchange/dipolar interactions in the YIG/Py bilayer define on-chip, magnetically distinct regions, effectively forming reflecting boundaries for magnons, resulting in a magnonic cavity. We confirm this by measuring multiple spin-pumping voltage (*V*^SP^) peaks with a 400 nm wide Pt strip placed along the center of the cavity. This voltage is proportional to the intensity of the FMR-excited magnon resonances in the *uncovered* YIG film, indicating the formation of standing-wave resonance modes. We assign these peaks to multiple standing backward volume spin-wave modes (BVSWs) and magnetostatic surface spin-wave modes (MSSWs), calculated from the spin-wave theory for a YIG slab with similar dimensions. Multiple spin-pumping voltage peaks were not observed for Pt strips placed outside the cavity. All of the measurements were performed at room temperature.

The presence of BVSWs and MSSWs modes in micrometer YIG cavities has already been observed.^37,38,41−44^ However, the YIG cavities were produced by a sputtering or wet-etching technique, and the modes were measured by a local FMR antenna or time-resolved magneto-optical Kerr effect. To the best of our knowledge, this work represents the first observation of cavity resonant modes measured by spin-pumping voltages using all-on-chip hybrid magnonic structures, illustrated in Figure 1a. The device was fabricated on a high-quality 100 nm thick YIG film grown by liquid phase epitaxy on a GGG substrate. Electron beam lithography was used to pattern the device, consisting of multiple strips of Pt and two Py squares with edge-to-edge distance of *w* = 2 μm, Figure 1b,c. The square shape was chosen to avoid effects of shape anisotropy in the Py film.^45,46^ The sample was placed on top of a stripeline waveguide and connected to a vector network analyzer. The stripline waveguide was then placed between two poles of an electromagnet in such a way that the DC external magnetic field, *B* = μ_0_*H*, where μ_0_ is the vacuum permeability, and the microwave field, *h*_rf_, were perpendicular to each other and both were in the plane of the YIG film in all the measurements; see Figure 1a,b. See Supporting Information section I for more details on sample fabrication and experimental setup.

**Figure 1:**
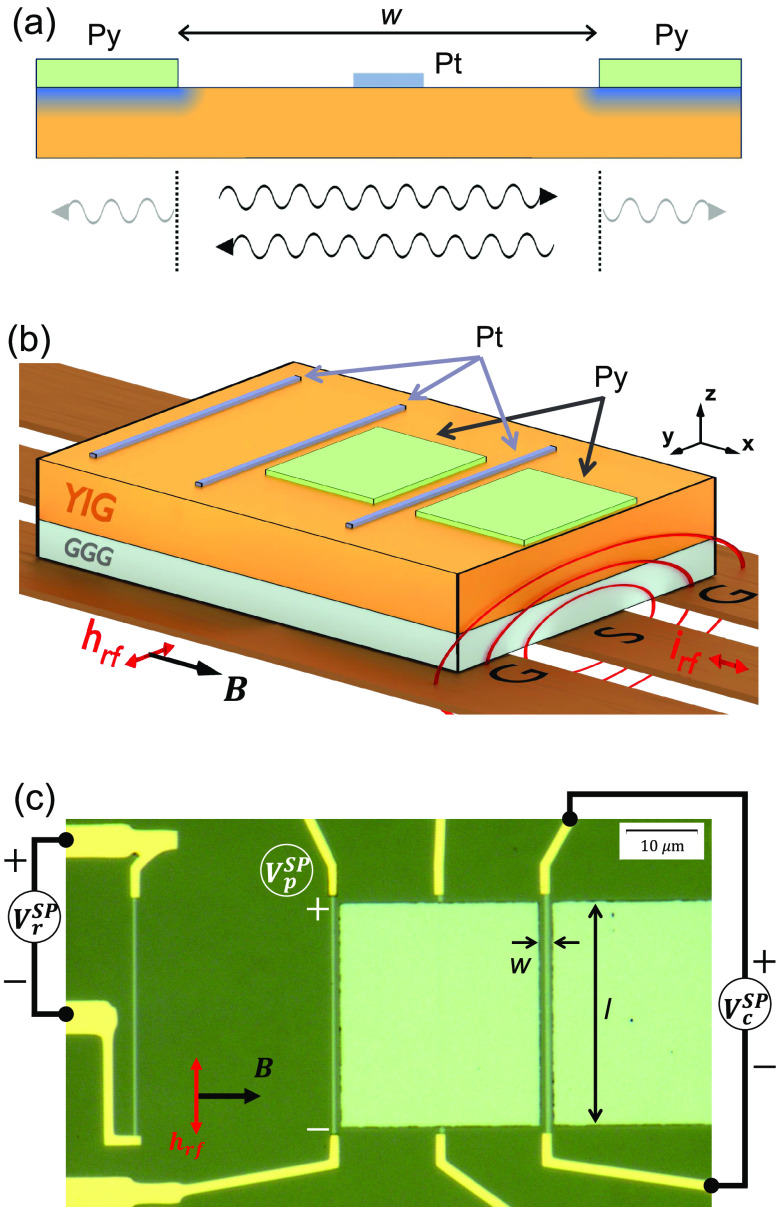
(a) Schematic illustration of the lateral view of the cavity formed by confining the YIG film between two YIG/Py bilayers. The Pt strip in the middle of the cavity can be used as a local magnon detector. (b) Schematic illustration of the waveguide stripline and sample arrangement of the microwave excitation setup for the FMR absorption and spin-pumping measurements. (c) Optical image of the fabricated devices and the electrical connections used in the experiments. The cavity is formed in the YIG region constrained between the Py squares, defined by *w* × *l*. Multiple peaks are observed with the platinum strip placed along the center of the cavity, *V*_c_^SP^, and absent in both Pt strips placed outside of the cavity, *V*_r_^SP^ and *V*_p_^SP^.

The *B*-field scan of the microwave absorption, S21, which measures the overall magnetic response of 4 × 3 mm sized YIG film, is shown in the top panel of Figure 2a, for 1–9 GHz. The FMR absorption peak of the 100 nm thick YIG film has a typical line width of Δ*B_FMR_* ≈0.2 mT, demonstrating the high quality of the YIG film. The FMR spectra are fit using an asymmetric Lorentizan function, obtaining the *B*-field value of the FMR peak and the line width (fwhm); see details in Supporting Information section I. The bottom panel of Figure 2a shows the microwave frequency versus the *B*-field value of the FMR peak. The solid blue curve corresponds to the best fit to the Kittel equation, ,^3^ where γ is the gyromagnetic ratio. The best fit was obtained for γ/2π = 27.2 ± 0.1 GHz/T and *M* = 142.4 ± 0.8 kA/m. The inset in Figure 2a shows the line width as a function of the microwave frequency. The solid blue curve corresponds to the best linear fit, obtaining a Gilbert damping of α ≈ 5.0 × 10^–4^ and the inhomogeneous line width of μ_0_Δ*H*_0_ = 0.06 mT. In this Letter, we address the uniform FMR resonance of the YIG film as “bulk” YIG resonance, or μ_0_*H*_FMR_, to distinguish the FMR absorption measurement of the mm-range size YIG film from the local spin-pumping voltage measurements for different platinum strips. The upper panel of Figure 2b shows the *B*-field scan of the spin-pumping voltage for the *remote* Pt strip, *V*_r_^SP^, for different microwave frequencies. The bottom panel presents the magnetic field of the *V*_r_^SP^ peak for different rf frequencies. Again, we fit the results using the Kittel equation, with γ/2π = 27.2 ± 0.1 GHz/T and *M* = 140.2 ± 0.9 kA/m.

**Figure 2:**
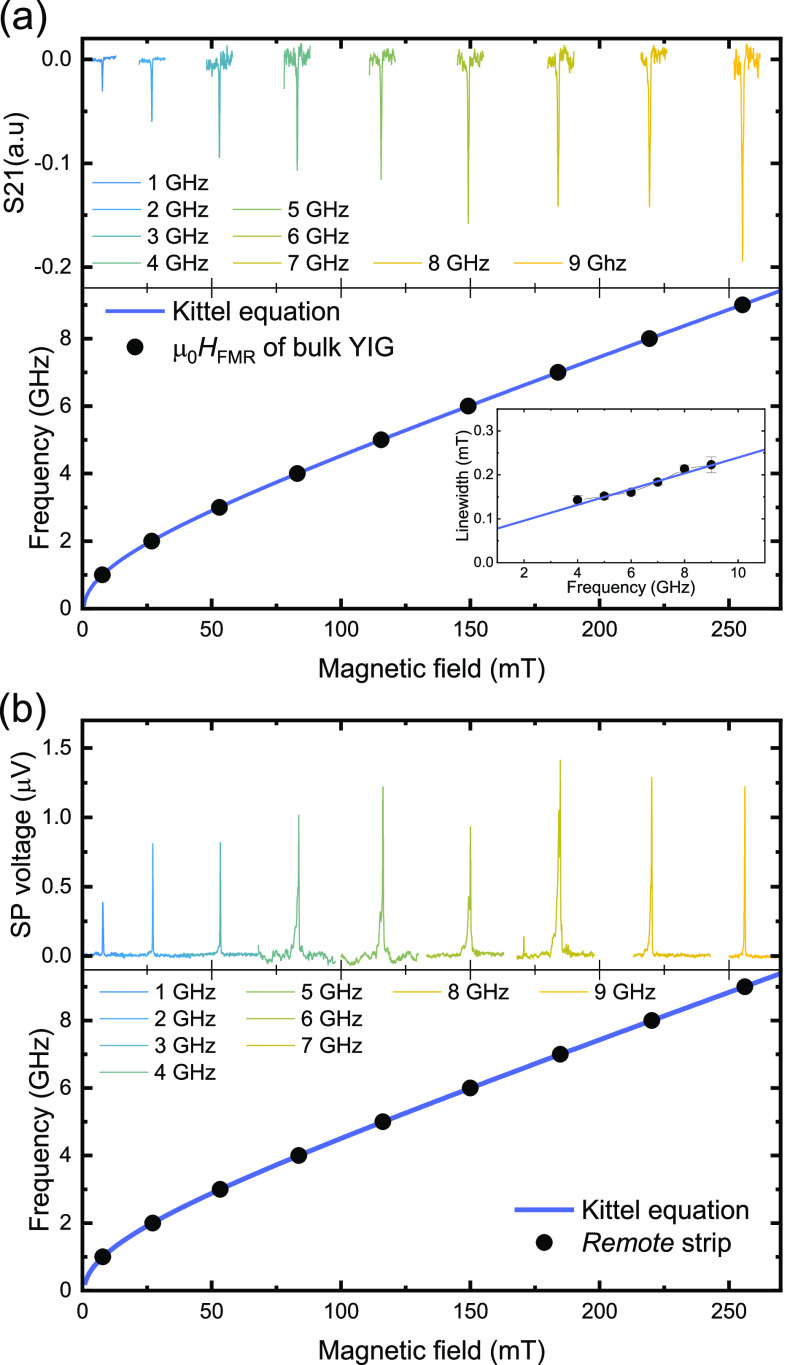
Comparison between FMR absorption of the bulk YIG and the spin-pumping voltage in the *remote* strip. (a) Top panel shows *B*-field scan of the microwave transmission absorption, S21. The bottom panel shows the field positions of FMR peaks for different microwave frequencies. The inset shows the line width vs resonance frequency. Note that we do not see the magnon spectra because the magnetic field is uniform on a relatively long scale. Therefore, only the uniform mode is excited and measured. (b) The top panel shows the *B*-field scans of the *V*^SP^ of the *remote* Pt strip for different microwave frequencies. The bottom panel shows the field position of the maximum *V*_r_^SP^ value as a function of the microwave frequency.

It is important to emphasize that although Figure 2 panels a and b look effectively identical, they correspond to two completely different experiments. Both measure the intensity of the ferromagnetic resonance, but in one case we measure the FMR absorption of the bulk YIG film, and in the other we measure the local spin-pumping voltage, as a result of the injection of spin current by means of the spin-pumping effect,^48^ and the conversion of the spin current into charge current in the Pt strip by the inverse spin Hall effect.^49,50^ We did not observe a significant peak broadening caused by the spin absorption due to the presence of the Pt layer. This is because the width of the strip is 400 nm, such that it covers only a fraction of the YIG film resulting in a spin-pumping response proportional to the FMR absorption of the bulk YIG.^51^ These results show that the platinum strip is a local and noninvasive intensity detector of the magnon excitation.

Figure 3 compares the *B*-field scan of the spin-pumping voltage measured on the *remote* strip, *V*_r_^SP^, solid black lines, and on the *cavity* strip, *V*_c_^SP^, solid blue lines, for different rf frequencies, from 2 to 9 GHz. The *remote* strip shows a single resonance peak, while the *cavity* strip shows multiple resonances. The average line width of the spin-pumping voltage peaks on the *cavity* strip is slightly broader than the *remote* strip, suggesting an additional damping contribution. Note that there is no pronounced peak on the *cavity* strip *B*-field scan corresponding to the bulk YIG resonance at 2 GHz, and the corresponding peak is small for 8 and 9 GHz, Figure 3a,e,f, respectively. This indicates that the spin-pumping voltage on the *cavity* strip is dominated by magnons excited inside the cavity itself and not by magnons generated outside of the cavity, corresponding to bulk FMR values, which could be transmitted into the cavity. As mentioned above, *V*_c_^SP^ is proportional to the resonance intensity of the YIG film in the region between the Py squares. This means that the series of resonance modes present underneath the Pt strip indicate the existence of a cavity supporting standing magnonic waves.

**Figure 3:**
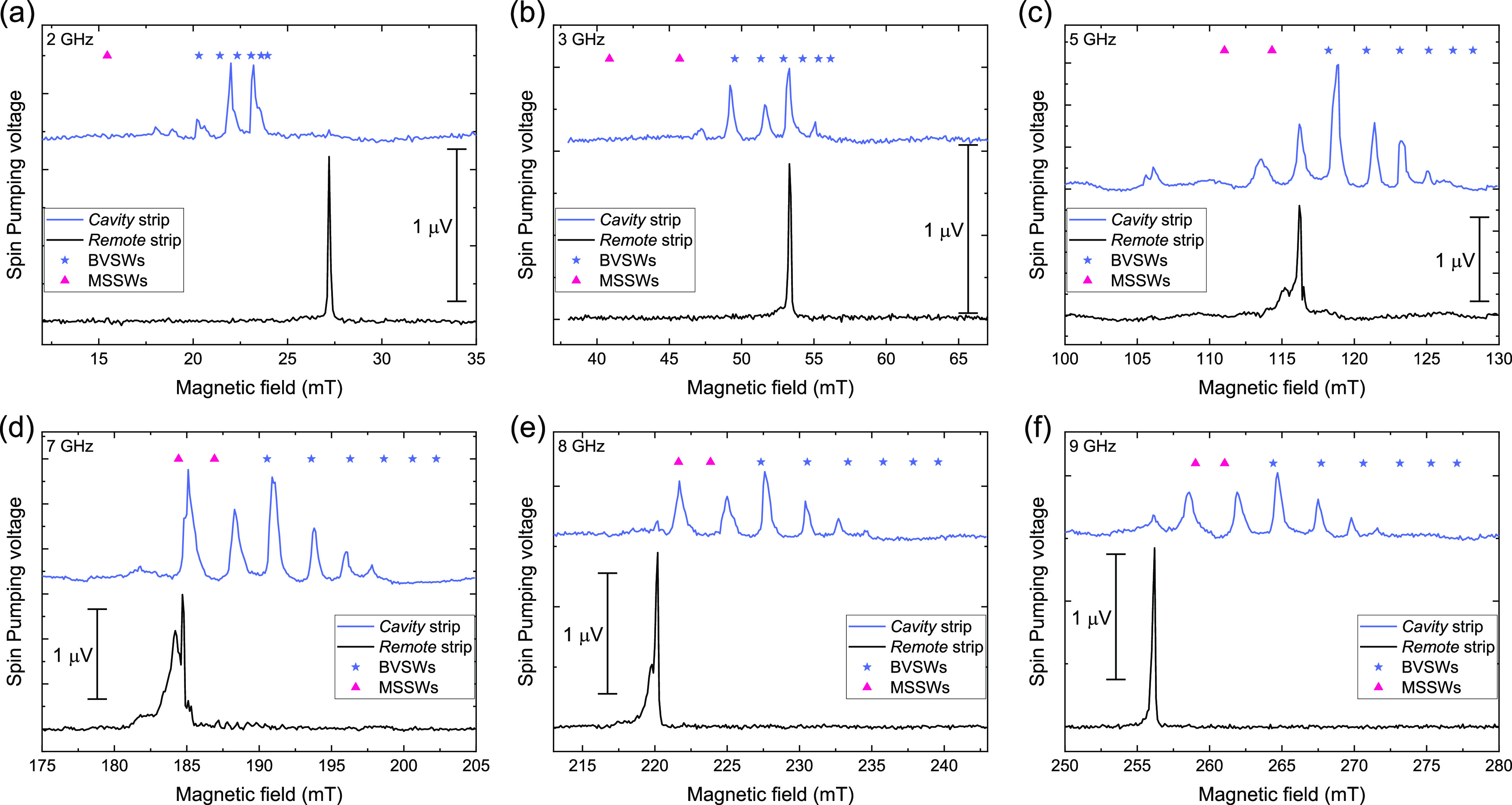
*B*-field scan of the spin-pumping voltage measured with the *remote* strip *V*_r_^SP^ and *cavity* strip *V*_c_^SP^ for different frequencies is shown from (a) to (f). The resonance modes of the *cavity* strip occur before the FMR of the bulk YIG resonance field for low frequency, 2 GHz, and are present after the bulk FMR resonance for 9 GHz. The resonance frequencies of the BVSWs and MSSWs modes obtained using eqs 1 and 2 are shown as blue star and pink triangle symbols, respectively, for each frequency. One can notice a secondary broad peak in the *remote* strip at 7 GHz. This peak is less evident or absent in other *remote* strip. We discuss more on that in the Supporting Information section III.

We can explain these modes by calculating the spin-wave dispersion relation for a YIG slab with dimensions *w* × *l* with magnetization in the plane of the film, given by^52−54^
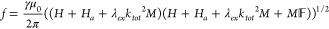
1where λ_*ex*_ = 2*A*/μ_0_*M*^2^ is the exchange constant with *A* = 3.5 pJ/m, *k*_tot_^2^ = *k*_*n*_^2^ + *k*_*m*_^2^ is the total quantized wavenumber defined by *k*_*n*_ = *nπ*/*w* and *k*_*m*_ = *mπ*/*l*, where *n* and *m* are the mode numbers along the width and length of the cavity, respectively. The function  can be written as
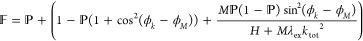
2where , for a YIG film with thickness *d*. In eq 2, ϕ_*k*_ = arctan(*k*_*m*_/*k*_*n*_), and ϕ_*M*_ is the angle between the magnetization and the spin-wave propagation or direction of k⃗. Two magnetostatic modes can be accessed considering the symmetry of the device, the magnetostatic surface spin-wave modes (MSSWs), where , i.e., ϕ_*M*_ = π/2 and the backward volume spin-wave modes (BVSWs) where , i.e., ϕ_*M*_ = 0.

The best fit to eqs 1 and 2 reproducing the majority of the spin-pumping peaks for different frequencies was obtained using *M* = 130 kA/m, *d* = 100 nm, *w* = 2.5 μm, *l* = 30 μm, μ_0_*H*_a_ = 10 mT, and γ/2π = 26.5 GHz/T. The calculated values for (*m* = 1) of the *n* = (1, 2, 3, ..., 6) mode of the BVSWs and first and second mode of MSSWs are shown in Figure 3a–f, blue star and pink triangle symbols, respectively. We obtained good agreement between the peaks present in *V*_c_^SP^ and the calculated modes. Since *l* ≫ *w*, modes with *m* > 1 are hard to distinguish since they are superimposed on the *m* = 1 mode due to their proximity in frequency. One important feature obtained from the fit is an anisotropy field of μ_0_*H*_a_ = 10 mT. The anisotropy may originate from the stray fields produced by the magnetization of Py, leading to a local increase of the effective DC-magnetic field applied on the cavity.^55^ Py stray fields may also be responsible for inducing even modes in the cavity, observed in our results.^55^ These even modes are not expected for a YIG slab when a homogeneous DC magnetic field and rf field are applied.^37,38^

One can see that the modes appear in Figure 3 at a lower field than the bulk YIG resonance for 2 GHz and a higher field than the bulk resonance at 9 GHz. We can analyze the frequencies of the cavity resonance modes by simultaneously plotting the SP-FMR resonance field of the *remote* strip (black circles) and the resonance field of each magnon mode in the *cavity* strip (vertical blue stripes), Figure 4a. Overall, the resonance field distribution of cavity modes evolves linearly in frequency, with a slope of ≈28 GHz/T, solid red curve in Figure 4a. The linear behavior was also reported in previous YIG cavity results.^38,43,56^

**Figure 4:**
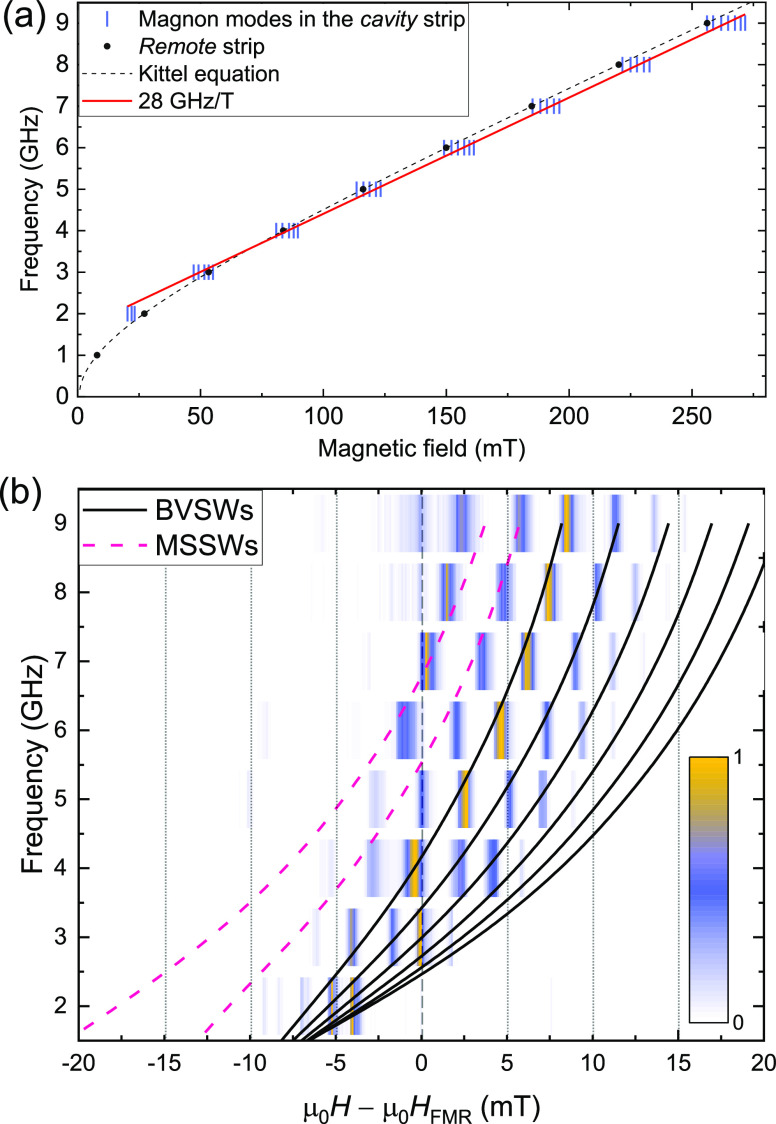
(a) Distribution of the identified magnon modes in the cavity as a function of the magnetic field for each frequency plotted as vertical blue stripes. The solid red curve corresponds to 28 GHz/T. The resonance of the *remote* strip and the best fit of the Kittel equation are addressed as a black symbol and a dashed black line, respectively. (b) Spin-pumping intensity spectra of the Pt strip within the cavity. The solid black and dashed pink lines correspond to the resonant modes for a YIG slab with dimensions similar to those of the build cavity.

To emphasize the localized magnon detection characteristic of the Pt strip, we normalize the spin-pumping voltage of the *cavity* strip, *V*_c_^SP^, between 0 and 1, based on the lowest and highest spin-pumping voltage value in each *B*-field scan. We centered the *B*-field scans with respect to the resonance field of the bulk YIG obtained from the FMR measurements, μ_0_*H*_FMR_, for each frequency. Figure 4b shows the dispersion of the resonant modes of the *cavity* strip compared with FMR of the bulk YIG. The BVSWs modes, up to *n* = 6, and the first and second modes of the MSSWs calculated from eqs 1 and 2 are shown in Figure 4b as solid black and dashed pink lines, respectively. The model of eqs 1 and 2 is very useful for confirming the mode position as a function of field and frequencies and the peak spacing between each peak. Presenting the voltages as intensity spectra demonstrates that the 400 nm wide Pt strip can be used as a localized FMR detector in future magnonic cavity studies. The spectrum at 1 GHz is not shown in Figure 4b due to the absence of a prominent spin-pumping voltage peak. Additional intensity spectra with other cavity widths are shown in the Supporting Information section II, confirming the reproducibility of the fabrication technique.

We do not observe multiple voltage peaks with the *proximity* strip, *V*_p_^SP^, placed close to the Py square but still outside the cavity. This confirms that the Pt layer alone does not induce sufficient magnetic changes in the YIG to create a cavity. Additionally, we do not observe multiple peaks in a device where the Py film was replaced with gold, ruling out microwave artifacts and confirming the requirement of confinement by YIG/Py bilayers on both sides to create the cavity; see Supporting Information section III. We hypothesize that the difference between the magnetization dynamics of covered and *uncovered* YIG regions by the Py film creates a magnon barrier, as illustrated in Figure 1a and discussed in previous reports.^28,29^

In analogy to a (lossy) cavity resonator, we can estimate a finesse given by Φ_*m*_ = Δ*B*_spacing_/Δ*B*, where Δ*B*_spacing_ is the *B*-field peak spacing and Δ*B* is the line width of the resonance peak.^57^ As a figure of merit, the frequency dependence of the average Δ*B*_spacing_ and calculated finesse is shown in Figure 5a. Although the peak spacing increases as a function of frequency, the finesse fluctuates around a value of Φ_*m*_ ≈ 10, about 1 order of magnitude higher than the previous report.^29^ This value of finesse corresponds to a reflectance of *R* ≈ 0.73, approximately constant through the entire frequency range. Figure 5b shows the average peak spacing and the finesse as a function of the cavity width, calculated from the *B*-field scans at 5 GHz; see Supporting Information section II. Although only three sets of data are presented, a clear correlation between the average peak spacing and finesse is observed. This correlation arises because the cavity is formed in a *uncovered* YIG region, preserving the optimal magnetic properties of YIG. In fact, the peak line width decrease with decreasing cavity width, *w*, indicating that the additional broadening may stem from the superposition of higher modes (*m* > 1) along the cavity length; see Supporting Information section II. The frequency spacing between these modes increases as a function of the aspect ratio (*l*/*w*). Ultimately, a maximum finesse of Φ_*m*_ ≈ 21, or *R* ≈ 0.86, is achieved for a cavity with *w* = 1.6 μm, which demonstrates a high potential for magnon confinement.

**Figure 5:**
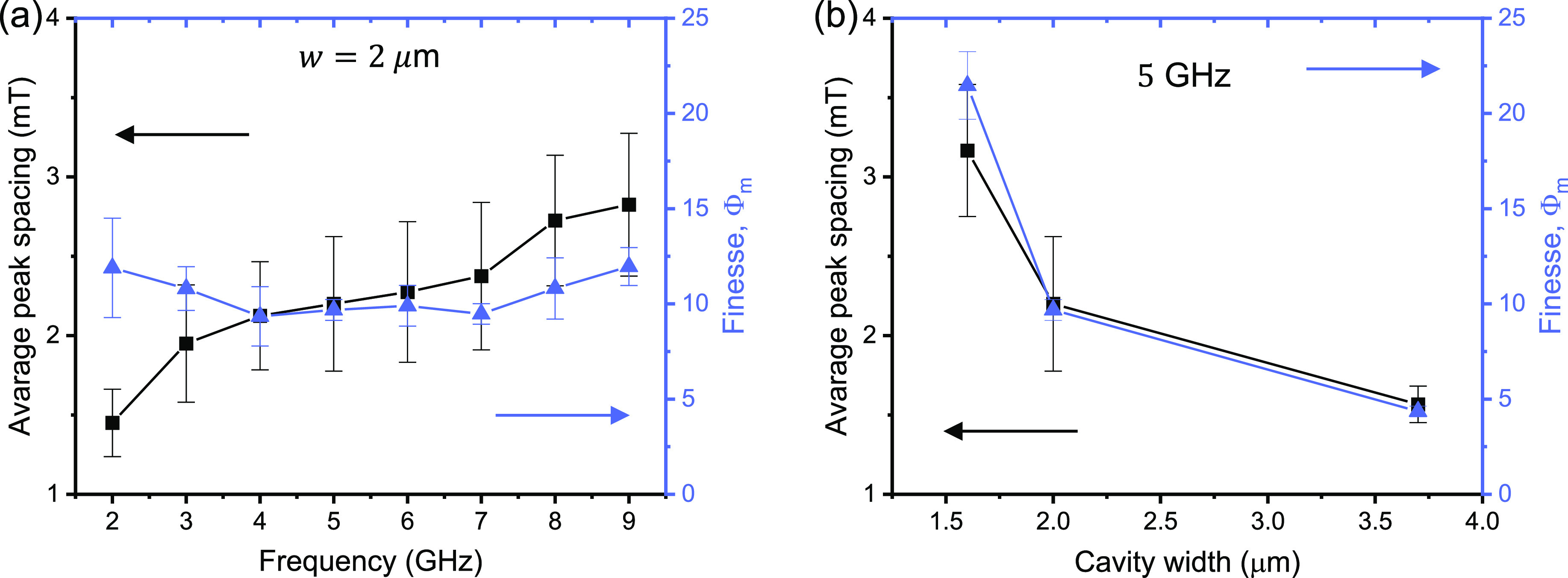
(a) Frequency dependence of the average Δ*B*_spacing_ and finesse Φ_*m*_. The error bar is calculated as the standard deviation of Δ*B*_spacing_. (b) Average peak spacing and finesse as a function of cavity width calculated from the *B*-field scan at 5 GHz.

We consider eqs 1 and 2 as an approximate model since it describes a YIG slab with dimensions *w* × *l*, where the magnetization amplitude is minimum at the boundary, i.e., an infinite potential well. In our case, the cavity is a consequence of the exchange and dipolar interaction in the YIG/Py bilayer.^7,8,29,58^ This means that a finite height potential well would better describe the system. This discrepancy can be the origin of a minor deviation between the measured *V*_c_^SP^ peaks and the calculated cavity modes. Accurate modeling of the modes should be performed using micromagnetic simulations, taking into account the exchange and dipolar interaction with Py in further investigation. The potential height of the cavity barriers should be dependent on the thickness of the YIG film and the exchange/dipolar interaction with the top ferromagnetic layer. Further investigation using the present technique should be performed for different adjacent ferromagnetic layers in which strong exchange interaction has already been reported, such as YIG/CoFeB^15^ and YIG/Co.^16^ YIG films thinner than 100 nm with the damping below 5.0 × 10^–4^ are good candidates to produce magnonic cavities with higher reflectance factors keeping the magnetic losses close to those reported in this letter.^59^

In summary, we take advantage of the difference in the magnetic dynamics between the YIG film and the YIG/Py bilayers to fabricate an all-on-chip magnonic cavity supporting standing magnon modes in a *uncovered* YIG film between two YIG/Py bilayers. This approach enables the confinement of magnons while preserving the optimal magnetic properties of the YIG cavity. The spin-pumping voltage of a 400 nm wide Pt strip proved to be a reliable technique to detect the magnon resonance modes of the cavity. Following this idea, 1D and 2D magnonic crystals could be obtained by having a regular array of magnetic strips onto YIG, with the possibility of measuring the magnon modes locally by means of spin-pumping. Moreover, further investigations should involve designing coupled cavities by placing two cavities side by side, where the coupling strength could be controlled by the width of the central YIG/Py bilayer. This cavity fabrication process opens new possibilities for investigating and characterizing micron-sized YIG cavities with a wide range of arbitrary shapes. It also allows for the implementation of on-chip magnonic computation structures, serving as a printed circuit board for magnons. These results demonstrate a promising combination of hybrid magnonics and cavity magnonics, which has the potential to drive the integration of future all-on-chip magnonic devices into mainstream microwave electronics.

## Supporting Information Available

Device fabrication and experimental setup details (section I), additional cavities measurements (section II), and control sample measurements (section III) (PDF)
