# Supplementary material for: Magnon Confinement in an All-on-Chip YIG Cavity Resonator Using Hybrid YIG/Py Magnon Barriers
Source: Nano Lett. 2023 Oct 11;23(20):9303–9. doi: 10.1021/acs.nanolett.3c02388 (PMC10603800; doi:10.1021/acs.nanolett.3c02388)
Supplement: Supplementary file 1 — nl3c02388_si_001.pdf [file nl3c02388_si_001.pdf]

SUPPORTING INFORMATION

Magnon confinement in an all-on-chip YIG cavity resonator using hybrid YIG/Py magnon barriers

Obed **Alves Santos***

Physics of Nanodevices, Zernike Institute for Advanced Materials,

University of Groningen, Nijenborgh 4,

Groningen, AG 9747, The Netherlands and

Cavendish Laboratory, University of Cambridge,

Cambridge, CB3 0HE, United Kingdom

Bart J. **van Wees**

Physics of Nanodevices, Zernike Institute for Advanced Materials,

University of Groningen, Nijenborgh 4,

Groningen, AG 9747, The Netherlands

I. SAMPLE FABRICATION AND EXPERIMENTAL SETUP

The samples consists of a high-quality 100 nm thick YIG film ($Y_3Fe_5O_{12}$) grown by liquid phase epitaxy on a GGG substrate, obtained commercially from Matesy GmbH, measuring approximately (4×3) mm. Electron beam lithography (EBL) was used to pattern the device, which consists of multiple strips of Pt with 35 μm length and 400 nm wide. The Permalloy (Py) squares have dimensions $(30 \times 30\ \mu m^2)$, the square shape was chosen to avoid shape anisotropies of the Py film.[1, 2] The Pt and Py layers were deposited by DC sputtering in an Ar+ plasma with thicknesses of 8 nm and 30 nm, respectively. The deposition of Ti(5 nm)/Au(75 nm) leads is made by e-beam evaporation. The last step consists of mounting the sample onto a non-resonant stripline waveguide, which can be used from 0.1 to 9 GHz with a characteristic impedance of 50 Ω, 0.030" RO4350, GCPWG, with a signal line 45 mil (1.14 mm) wide. Figure S1 a) show an optical image of the final YIG film sample containing multiple devices, while b) shows a zoom-in image of an individual device. The wire bonding is made using AlSi (Al 99%, Si 1%) wires on the sample holder and connected to a lock-in amplifier.

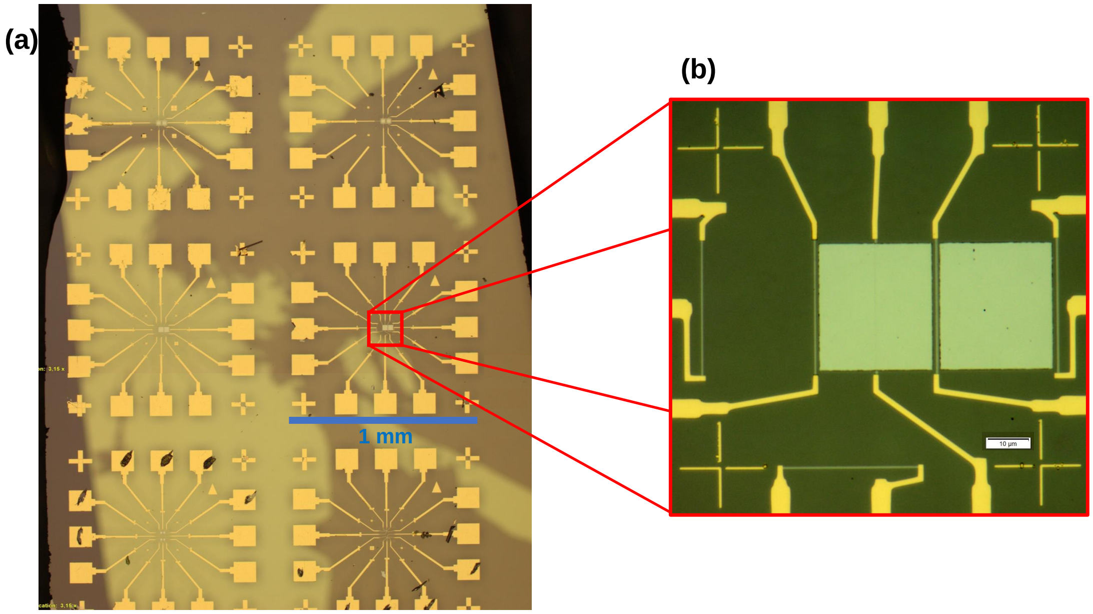

FIG. S1. **a)** Show the optical image of the final YIG sample with multiple devices. **b)** Show a zoom-in optical image of an individual device.

* **Correspondence should be addressed:**

oa330@cam.ac.uk, or obed.alves.santos@gmail.com

The microwave field excitation is sent connecting the stripline waveguide to a vector network analyzer (VNA). The FMR absorption of the YIG film is obtained by scanning the absorption (S21) or reflection (S11) in the VNA for a fixed microwave frequency as a function of the magnetic field. To obtain the $\mu_0 H_{\mathrm{FMR}}$ value and the FMR linewidth (ΔH), each S21 B-field scan is fitted using an asymmetric Lorentzian function, $L(H-H_{FMR}) = S\Delta H^2/[(H-H_{FMR})+\Delta H^2]+A[\Delta H(H-H_{FMR}]/[(H-H_{FMR})+\Delta H^2]$. Where S and A are the symmetric and antisymmetric amplitudes. The spin pumping voltage is measured at the end of the Pt strips by a lock-in amplifier, triggered with the VNA. The microwave is then switched from low power $P_{rf}^{low} = -25$ dBm to high power $P_{rf}^{high} = 16$ dBm, with a modulation frequency of 27.71 Hz. The (waveguide stripline + sample) is positioned between two poles of an electromagnet such that the external magnetic field (H) and the microwave field (h_{rf}) are perpendicular to each other, and both are in applied in the plane of the YIG film. All the measurements were performed at room temperature.

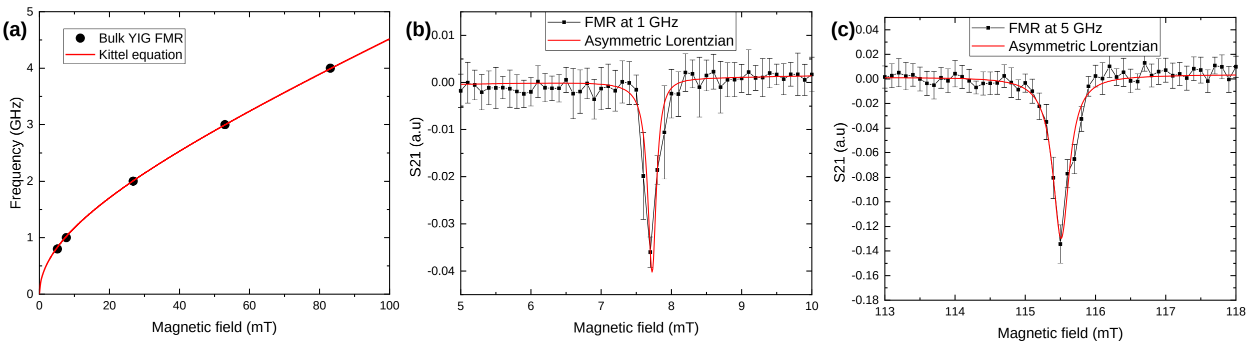

FIG. S2. **a)** Field resonances of the bulk YIG FMR for low frequency. **b)** and **c)** are S21 measurements of the bulk YIG FMR at 1 GHz and 5 GHz, respectively. Our external magnetic field step corresponds to 0.1 mT. The error bar corresponds to the standard deviation from the multiple VNA readings.

In spintronics, the FMR drives the spin pumping effect (SPE),[3] where a flow of spin current, occurs from the ferromagnetic/ferrimagnetic layer towards an adjacent layer at the peak of the microwave absorption during the FMR process.[4] Therefore, the YIG film injects a spin current by means of the SPE into the Pt strip.[3] That spin current can be expressed as

$$J_s = g_{eff}^{\uparrow\downarrow} \frac{\hbar\omega}{4\pi} \left(\frac{h_{rf}}{\Delta H}\right)^2 L(H-H_{FMR}), \tag{S1}$$

where $g_{eff}^{\uparrow\downarrow}$ is the effective spin mixing conductance, $\omega = 2\pi f$ is the rf frequency, $\hbar$ is the reduced Planck constant, and $L(H-H_{FMR})$ is the FMR absorption, usually a Lorentizian-like line shape. The spin current along $\hat{z}$ with spin polarization $\vec{\sigma}$ along $\hat{x}$ is converted into charge current along $\hat{y}$

direction by the inverse spin Hall effect (ISHE), following $\vec{J}_c \propto \theta_{Pt}(\vec{J}_s \times \vec{\sigma})$.[5] Where θ_{Pt} is the spin Hall angle, which quantifies the conversion efficiency between spin and charge currents. The total voltage build-up at the edge of the Pt strip can be expressed by[5, 6]

$$V^{SP} = \theta_{Pt} \frac{R_{Pt}\lambda_{Pt}w_{Pt}}{t_{Pt}} \left(\frac{2e}{\hbar}\right) \tanh\left(\frac{t_{Pt}}{2\lambda_{Pt}}\right) J_s, \tag{S2}$$

where R_{Pt}, t_{Pt}, w_{Pt} and λ_{Pt} are the resistance, thickness, width, and the spin diffusion length of the Pt strip.

The spin pumping process typically leads to an increase in the FMR linewidth as it introduces an additional component to the magnetic losses of the ferromagnetic layer. However, in our measurements, we did not observe any significant broadening of the linewidth in the spin pumping voltages measured on the Pt strip. This absence of pronounced linewidth broadening in V_r^{SP} can be attributed to the fact that the Pt strip only covers a fraction of the YIG film. Cheng *et al.* demonstrated that for Pt strips with widths $w < 5$ μm, the spin pumping voltage is dominated by the spin current injected in the proximity of the Pt strip, within a YIG region unaffected by the presence of the Pt layer. In this region, the damping and linewidth correspond to the bulk values. Hence, the spin pumping voltage measured with the Pt strip effectively acts as a localized FMR absorption detector or an rf antenna.[7]

One can realize that an additional spin pumping voltage could be detected by the Pt strip within the field range corresponding to the resonance of the Py layer, resulting from spin pumping from Py towards the YIG film. However, in our measurements, we observed that the voltage remained below the noise level throughout the resonance field of Py. As a result, our main focus in this study is on the resonance of the YIG film.

II. ADDITIONAL CAVITIES MEASUREMENTS

Additional spin pumping measurements were conducted on several other cavities to demonstrate the reproducibility of the method. Optical microscope images of the cavities with different widths are shown in Figure S3 a) and b). The main results presented in the text are based on the cavity denoted as "Cavity A," which has a width of $w = 2.0$ μm. Subsequently, we present results for two separate cavities: "Cavity B" and "Cavity C," both with a width of $w = 1.6$ μm, as well as "Cavity D", which has a width of $w = 3.7$ μm. Figure S3 c) shows the average peak spacing, average peak linewidth, and finesse as a function of the inverse square of the cavity

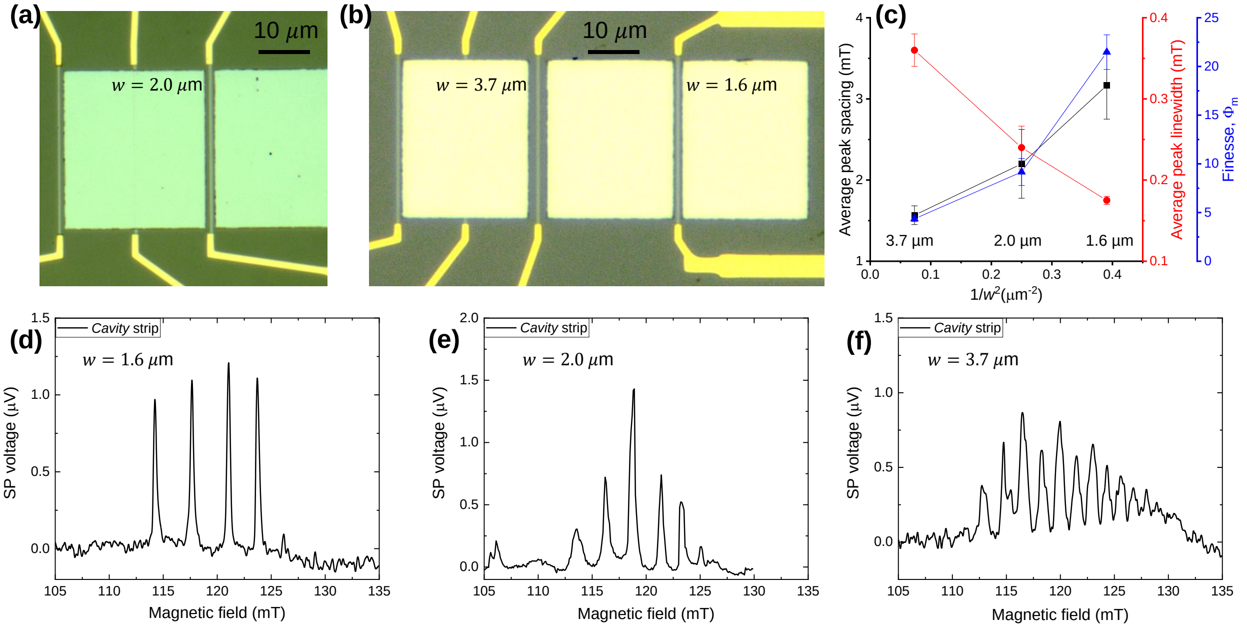

FIG. S3. **a)** and **b)** show optical images from different devices fabricated with distinct distance cavity widths, indicated in the figure. **c)** Average peeks spacing in (mT), average peak linewidth, and calculated finesse as a function of the inverse square of the cavity width (w). **d)**, **e)** and **f)** shows B-field scan of the spin pumping voltage in the central platinum strip at 5 GHz, for a cavity width of $w = 1.6\ \mu$m, $w = 2.0\ \mu$m, and $w = 3.7\ \mu$m, respectively.

width. It can be observed that the peak spacing shows a linear dependence as function of $1/w^2$ within the error bar, following the expected behavior for a magnonic cavity.[8, 9] The linear behavior is also evident in the average peak linewidth, suggesting that increasing the aspect ratio of the cavity (l/w) leads to a more homogeneous distribution of cavity modes. However, it should be noted that our results are limited to only three different cavity widths, making it challenging to make a definitive statement about the observed behavior. Figure S3 d) to f) show the B-field scan of the spin pumping voltage for three cavities widths for 5 GHz. As the width decreases, a few observations can be made. Firstly, the peak linewidth decreases, indicating a narrower spectral distribution of the spin pumping voltage. Secondly, the peak spacing increases, suggesting a larger separation in corresponding excitation frequencies. Finally, the spin pumping voltage height becomes more equal for the cavity with $w = 1.6\ \mu$m.

Figure S4 a) to c), shows the B-field spin pumping voltage for the *remote* cavity and cavities B and C. All three measurements were obtained simultaneously, with each *cavity* strip connected to an independent lock-in amplifier. Both cavities, B and C, have been designed with the same

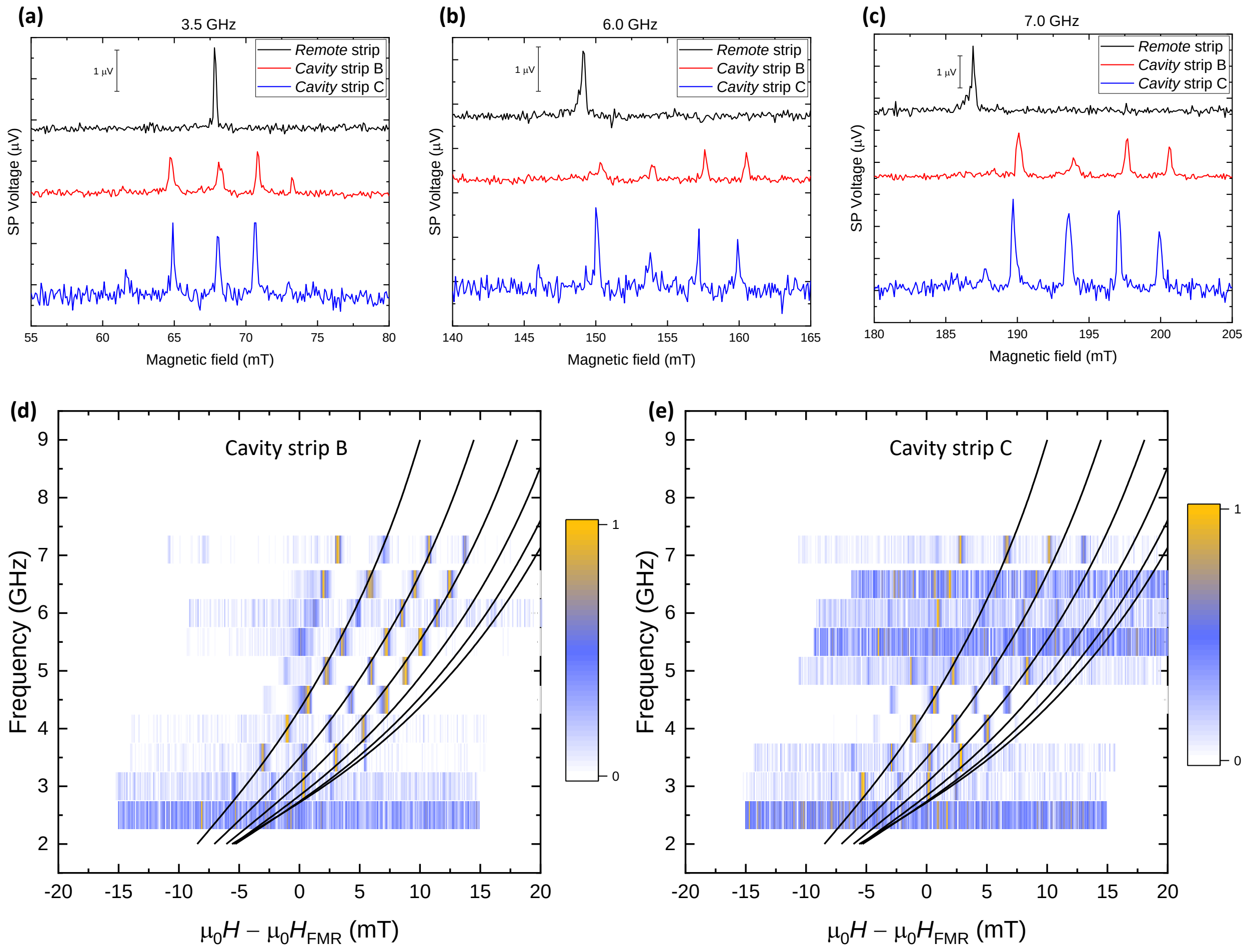

FIG. S4. **a)** to **c)** shows the B-field scan of the spin pumping voltage obtained for the *remote* strip and the Pt strips placed along the center of the cavity B and C, respectively. **d)** and **e)** shows the spin pumping intensity spectra for two different cavities with the same width distance of $w = 1.6\ \mu$m.

width of $w = 1.6\ \mu$m. This explains why the field position of almost every peak aligns with each other. Once again, it is evident that the corresponding peak for the bulk YIG resonance is absent or reduced to the noise level at 6 GHz and 7 GHz. This observation highlights that the voltage peaks are primarily determined by the magnon modes confined within the cavity. One can also observe a small alternation of the intensity between consecutive peaks in Figure S4 a) to c). A similar trend seams to be present in Figure 3 of the main text. This behavior might occur due to the difference in microwave excitation corresponding to odd and even modes. However, we cannot explain them in detail without more detailed micromagnetic simulations, taking into account the exchange and dipolar interactions in the YIG/Py bilayer, and then correctly addressing each peak to the corresponding magnon mode and intensity.

The spin pumping intensity spectra for cavities B and C are present in Figure S4 d) and e), with a frequency spacing of 0.5 GHz. Although the peak between 5.5 GHz and 6.5 GHz in cavity C is difficult to identify due to the noise, both cavities exhibit a similar peak dispersion, with almost every peak matching at each frequency. The solid black lines in Figure S4 d) and e) are the modes calculated by equation (1) and (2) from the main text, up to $n = 6$ using the following parameters: $w = 1.6\ \mu$m, $l = 30\ \mu$m, $M = 130$ kA/m, $\mu_0 H = 14$ mT, and $\gamma/2\pi = 26.5$ GHz/T. We believe the small diminishment in the gyromagnetic ratio could be due to other factors and effects not included in the model, such as the boundary interface with the YIG/Py bilayer. These results confirm the reproducibility of the technique.

III. CONTROL SAMPLES

In Figure 3 d) on the main text, one can identify a secondary peak at 7 GHz. This secondary "peak" at the left side of the spin-pumping-FMR peak might be caused by the excitation of the finite k bulk-magnons, corresponding to the spin wave modes in the whole YIG sample. The corresponding peak is also not observed in the FMR absorption. This secondary structure is less evident or absent in another remote Pt strip using a different YIG sample, shown in Figure S5, as well as in Figure S6. We believe this structure could be due to the magnon interferences in the bulk-YIG region. This does not affects the analysis of the resonance modes of the cavity.

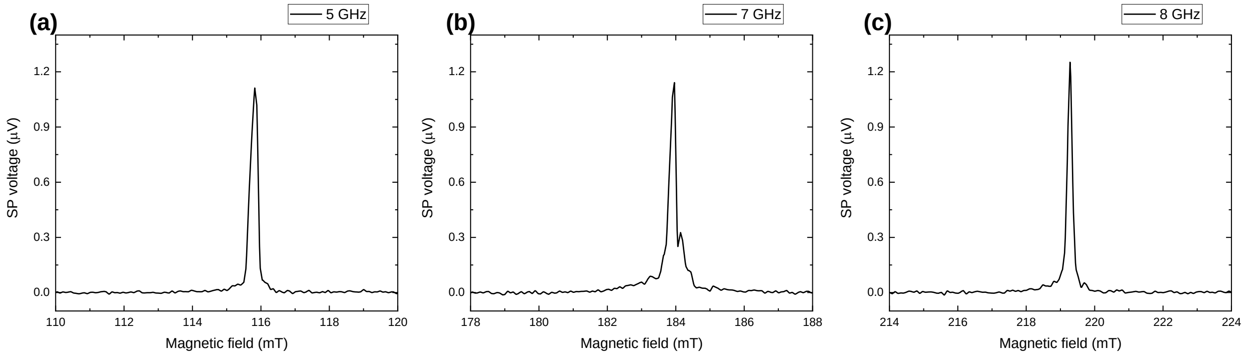

FIG. S5. Less intense or not present "secondary" peak in a *Remote* Pt strip on a different 100 nm thick YIG sample at **a)** 5 GHz, **b)** 7 GHz, and **a)** 8 GHz. Further experiments using rectangular microwave waveguides may avoid surface mode excitations in the bulk YIG film.

Figure S6 a) shows the absence of magnetostatic modes in a control device where an Au film replaces the Py film. This result rules out the possibility of (multiple) peaks originating from

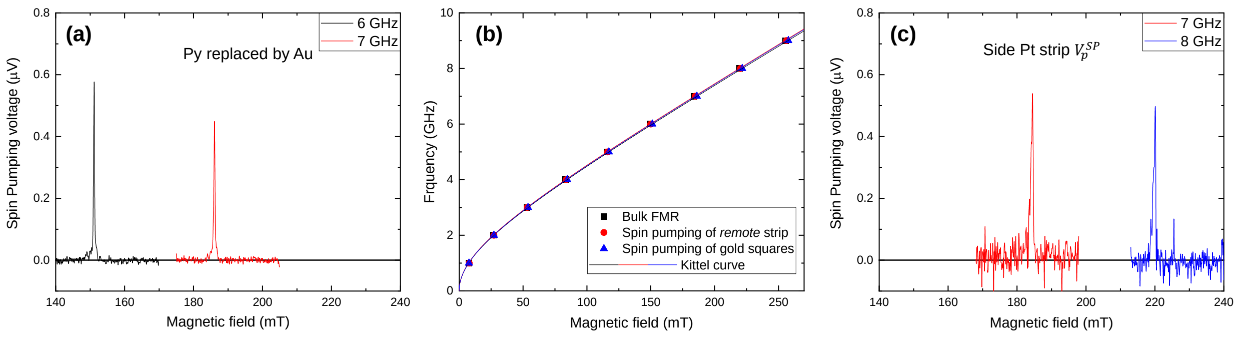

FIG. S6. **a)** Absence of the multiple peaks when the Py squares are replaced with gold. **b)** Comparison of Kittel fitting between FMR of YIG film, spin pumping voltage in *remote* strip, and when Py square is replaced with Au. **c)** Spin pumping voltage with a *proximity* strip, no evidence of cavity resonant modes was observed.

microwave artifacts caused by the proximity of a metallic film to the Pt strip.. Figure S6 b) shows the direct comparison of the Kittel equation for FMR of the YIG bulk, the spin pumping voltage of the *remote* Pt strip, and the spin pumping voltage of the Pt strip when Py is replaced with gold. The adjusted Kittel curves show only a slight deviation. These results also suggest that the magnetic nature of the exchange/dipolar interaction between the YIG and Py films is crucial for constructing the cavity through engineered-design lithography.

The spin pumping voltage was also measured in a Pt strip located near the left side of the Py square, V_p^{SP} in Figure 1 b) of the main text. However, despite the higher noise level, the spin pumping voltage as a function of the B-field did not exhibit any evidence of (multiple) peaks, as shown in Figure S6 c). It becomes evident that the presence of YIG|Py interfaces on both sides is necessary to create the magnon resonant modes within the cavity. In Figure 1 c) of the main text, one can identify a Pt strip placed underneath the middle of the Py square. The electrical resistance of that strip was in the MΩ range, much higher than the usual 3.5 $k\Omega$ for 400 nm wide, 35 μm long, and 8 nm thick Pt strip. This suggests that the Pt strip may have been damaged during the sputtering and lift-off process involved in the fabrication of the Py square. As a result, we do not have reliable data for the electrical contact of that particular strip.

---

[1] M. Mruczkiewicz, P. Graczyk, P. Lupo, A. Adeyeye, G. Gubbiotti, and M. Krawczyk, Spin-wave nonreciprocity and magnonic band structure in a thin permalloy film induced by dynamical coupling

with an array of Ni stripes, Physical Review B **96**, 104411 (2017).

[2] A. Talapatra and A. Adeyeye, Linear chains of nanomagnets: engineering the effective magnetic anisotropy, Nanoscale **12**, 20933 (2020).

[3] Y. Tserkovnyak, A. Brataas, and G. E. Bauer, Enhanced gilbert damping in thin ferromagnetic films, Physical Review Letters **88**, 117601 (2002).

[4] S. Mizukami, Y. Ando, and T. Miyazaki, Ferromagnetic resonance linewidth for NM/80NiFe/NM films (NM= Cu, Ta, Pd and Pt), Journal of Magnetism and Magnetic Materials **226**, 1640 (2001).

[5] J. Sinova, S. O. Valenzuela, J. Wunderlich, C. Back, and T. Jungwirth, Spin Hall effects, Reviews of Modern Physics **87**, 1213 (2015).

[6] A. Azevedo, L. Vilela-Leão, R. Rodríguez-Suárez, A. L. Santos, and S. Rezende, Spin pumping and anisotropic magnetoresistance voltages in magnetic bilayers: Theory and experiment, Physical Review B **83**, 144402 (2011).

[7] Y. Cheng, A. J. Lee, G. Wu, D. V. Pelekhov, P. C. Hammel, and F. Yang, Nonlocal uniform-mode ferromagnetic resonance spin pumping, Nano Letters **20**, 7257 (2020).

[8] T. Yu and G. E. W. Bauer, Efficient gating of magnons by proximity superconductors, Physcal Review Letters **129**, 117201 (2022).

[9] Y. W. Xing, Z. R. Yan, and X. F. Han, Magnon valve effect and resonant transmission in a one-dimensional magnonic crystal, Physical Review B **103**, 054425 (2021).
